# Nutritional Status of Children with Newly Diagnosed Food Allergies

**DOI:** 10.3390/children10101687

**Published:** 2023-10-14

**Authors:** Martyna Jasielska, Anna Buczyńska, Piotr Adamczyk, Urszula Grzybowska-Chlebowczyk

**Affiliations:** Department of Pediatrics, Faculty of Medical Sciences in Katowice, Medical University of Silesia, 40-752 Katowice, Poland; anna.buczynska@sum.edu.pl (A.B.); padamczyk@sum.edu.pl (P.A.);

**Keywords:** food allergy, children, malnutrition

## Abstract

Background: Most published pediatric guidelines on food allergy highlight the importance of nutritional counseling and dietary adequacy to avoid either growth retardation or nutritional deficiencies. The aim of the study was an assessment of the nutritional status of children with IgE-mediated food allergies. Material and method: 45 patients with newly diagnosed food allergy (FA) and 33 healthy controls were analyzed (aged 6 to 72 months, 60.2% boys). The nutritional status was assessed using anthropometric measurements (body weight and length) and serum laboratory tests. The results were analyzed with the Statistica 12 software (Tulsa, OK, USA). Results: 82%, 40%, 8.8%, and 6.6% of the studied children demonstrated allergy to hen’s egg, cow’s milk, pork meat, and wheat/rye, respectively. Z-score BMI < −2SD was more often found in the FA subjects under 30 months of age than in the controls (*p* = 0.04). As many as 77.8% of the FA subjects and 78.8% of the controls were of normal height (hSDS: −0.23 ± 1.74 and −0.31 ± 1.49, respectively, *p* = 0.8). Retinol binding protein four serum concentration was significantly lower in the FA group (17.01 ± 3.84 mg/L) than in the controls (20.47 ± 4.87 mg/L, *p* < 0.001). No statistically significant differences were observed between the FA group and the controls (either in the younger or the older age group) (*p* > 0.05) for the serum concentrations of total protein, total cholesterol, thyroxin-binding prealbumin (TBPA), 25(OH)D, hemoglobin level or white blood cells. Conclusions: In patients under 30 months of age, one of the symptoms of food allergy may be body weight deficiency, while short stature is less common at the time of diagnosis.

## 1. Background

Current studies suggest that food allergies are a serious and common health problem. In some countries, up to 10% of children are affected. In European children below the age of 5 years, the prevalence rates of food allergy have been reported from 3.6% (in Denmark) to 6.8% (in Norway) [1]. Additionally, the number of pediatric patients with food allergies has been increasing in recent decades, especially in children from highly developed countries [1,2]. Eggs, peanuts, cow’s milk, soy, nuts, shellfish, fish and meat, or wheat are the most common food allergens, being responsible for more than 90% of identified food allergies [1,3]. The symptoms of a food allergy may affect the digestive system, the skin, or the respiratory system. Gastrointestinal symptoms include regurgitation and vomiting, diarrhea, constipation, or intestinal colic [4].

Milk, dairy products, and eggs are important components of the diet in early childhood. The most effective way of food allergy control is to eliminate the allergen(s) from the diet. On the other hand, a poorly conducted elimination diet may exert adverse effects, such as malnutrition and/or growth retardation. Additionally, about 30% of food-allergic patients in the U.S.A. reveal multiple food allergies, where the most common allergens are found in milk, peanuts, and shellfish [4]. The exclusion of important nutrients from diet is just one of the causes of the abnormal development of children with food allergies. An equally important element may be the increased requirements due to inflammation, associated feeding disorders, or increased losses of nutrients via persistently inflamed gastrointestinal mucosa and skin—these may also be the mechanisms underlying the insufficient nutritional intake in food-allergic children [5].

Previous studies have shown that children with food allergies and those on an elimination diet are more likely to suffer from body height and weight deficiency. The incidence of body weight deficiency increases with the growing number of eliminated products [6,7].

## 2. Objectives

The majority of pediatric nutritional guidelines emphasize the importance of nutritional counseling and a balanced diet to avoid growth retardation or nutritional deficiencies. Considering the lack of publications evaluating pediatric patients with food allergies at the moment of diagnosis, this study was planned to analyze anthropometric measurements and laboratory parameters of the nutritional status in children with food allergies juxtaposed to healthy controls. The primary aim of the study was an assessment of the nutritional status of children with IgE-mediated food allergies.

## 3. Materials and Method

The analysis included 78 patients aged from 6 to 72 months (the mean age: 31.8 months); 60.2% were boys. The food-allergic patients (FA, *n* = 45 children) were diagnosed and enrolled in the study at a single regional medical center for pediatric gastroenterology (the Department of Pediatrics, Medical University of Silesia, Katowice, Poland) in the years 2018–2019. Food allergies were diagnosed based on clinical symptoms (convincing history of allergic reactions) and by serum-specific IgE measurements. Specific IgE was assayed using the Immulite 2000 3gAllergy(TM) Specific IgE test. The reporting range was 0.10–100 IU/mL, and the quantitative result in IU/mL was simultaneously assigned to one of 0–6 classes of the RAST scale. The specific IgE antibody (sIgE) values of at least 0.7 IU/mL to food allergens were considered positive (class 2 of the RAST scale). The following specific antigens were taken into consideration: egg white, egg yolk, cow’s milk, whey, casein, pork, chicken and beef meat, wheat, rye, corn, rice, and soybean. Initial diagnostic procedures were performed during the presentation of clinical symptoms and before any modifications of diet or pharmacological treatment were employed. Allergy was confirmed by food elimination for 7–14 days, followed by an open challenge test.

The control group (C, *n* = 33) consisted of healthy children with excluded food allergies (based on patient medical history, the absence of food allergy symptoms, and negative sIgE). The study participants were also divided according to age into a younger group, with subjects up to 30 months of age, and an older group with children above 30 months of age. Study groups showed no statistically significant differences in terms of age and sex with the corresponding control groups (*p* > 0.05).

All the patients with organic gastrointestinal diseases (including celiac disease and inflammatory bowel disease) and the patients with eating disorders were excluded from the study.

The nutritional status was assessed at the time of allergy diagnosis, before the introduction of a permanent elimination diet. All the patients were assessed using anthropometric measurements (body weight and length/height) and laboratory tests performed on fasting blood samples (blood was collected at the same time for all the tests described). Body weight and length (<24 months) or height (>24 months) were measured using standard procedures. The measured body weight and height values were assessed, according to the Polish standards, using the anthropometric indicators of the BMI-Z-score and height standard deviation score (hSDS), the length Z-score (lZ-score), and the height Z-score (hZ-score) [8]. The results of hSDS ≤ −2 SD were considered as short stature. Body mass deficiency was diagnosed when the BMI Z-score was ≤−2 SD. According to recommendations, SD ≥ 1 and SD ≥ 2 were interpreted as overweight and obesity, respectively [8].

Complete blood count (CBC), plasma 25-hydroxyvitamin D (25[OH]D), total cholesterol (TC), ferritin, total protein, and albumin levels were assayed using standard methods both in the patients and in the controls. Retinol-binding protein 4 (RBP) and thyroxin-binding prealbumin (TBPA) assays were performed using the ELISA method. Serum 25[OH]D was assessed using the electrochemiluminescent method (Elecsys Vitamin D total, Cobas, Indianapolis, IN, USA). Serum 25[OH]D deficiency was set as either less than or equal to 20 ng/mL, with insufficiency defined as 21 to 29 ng/mL.

A statistical analysis was carried out using the Statistica 12 software (StatSoft, Tulsa, OK, USA). The mean values and standard deviations were used for descriptive statistics of continuous variables. The normality of data distribution was verified using the Shapiro–Wilk test. Absolute and percentage values were provided for qualitative variables. The Student’s *t*-test for independent samples or the Mann–Whitney U test was applied for the comparative analyses of continuous variables, depending on data distribution. Comparisons of qualitative feature prevalence rates were performed using the chi-square test. The significance of results in all the statistical analyses was assumed at *p* < 0.05.

All the study methods were carried out in accordance with the relevant guidelines and regulations. The protocol of the study was approved by the Bioethical Committee of the Medical University of Silesia in Katowice, Poland, No. KNW/0022/KB1/2/18. The study subjects and/or their parents or legal guardians provided informed consent.

The study project was financed by the Medical University of Silesia, Katowice, Poland. Grant numbers [KNW-1-070-N/8/K and KNW-1-172/K/9/K].

## 4. Results

The group of food allergic (FA) patients consisted of 45 children (64.4% were boys), including 19 children under 30 months of age (FA1) and 26 children aged above 30 months (FA2). A total of 33 healthy children were included in the control group (54.5% were boys), 21 children were under 30 months of age (C1 group), and 12 children were in an older group (C2).

The clinical presentation of food allergy cases included atopic dermatitis in 23 patients (51%), abdominal pain in 18 patients (40%), constipation in 7 patients (15.5%), and diarrhea in four patients (8.8%). Only one patient suffered from bronchial asthma (2.2%). In total, 42% of FA patients demonstrated symptoms from more than one system.

Out of all the children with allergies, there were 82% children with allergy to hen’s egg, 40% to cow’s milk, 8.8% to pork, and 6.6% to wheat and rye. Among the FA1 subjects, allergy to hen’s eggs concerned 17 patients (89.4%), and allergy to cow’s milk was observed in 9 children (47.3%). In the FA2 group, the most common allergy was also that to eggs, affecting as many as 21 patients (80.7%), and to cow’s milk or its antigens diagnosed in 9 patients (34.6%). The highest allergy classes were observed for egg, milk, and soy. In one patient only were the symptoms of allergy classified as severe (anaphylactic reaction). Detailed data are presented in Table 1.

There was no significant difference between the FA subjects and the controls regarding the BMI Z-scores, either for the whole study group or for the particular age subgroups (*p* > 0.05). However, in the FA1 subgroup, body weight deficiency (BMI Z- score < −2SD) was more often identified than in the controls (60% vs. 23.8%, respectively, *p* = 0.04). No such difference in the prevalence of underweight was observed in the older age subgroup (FA2) (*p* > 0.6). BMI Z-score > 1 SD was found in 11.1 and 12.1% of the FA patients and the controls, respectively.

As many as 77.8% of the FA subjects and 78.8% of the controls were of normal height (hSDS: −0.23 ± 1.74 and −0.31 ± 1.49, respectively *p* = 0.8). Additionally, when the age subgroups were compared, no statistically significant differences were found in the prevalence of short stature (*p* > 0.05).

The demographic characteristics, anthropometric measurements, and laboratory parameters of the groups, divided according to age category, are presented in Table 2 (the younger children) and Table 3 (the older children).

RBP serum concentrations were significantly lower in the whole FA group (17.01 ± 3.84 mg/L) than in the controls (20.47 ± 4.87 mg/L, *p* < 0.001). However, taking into account the age criterion, that difference was statistically significant only in the older group of patients (*p* = 0.02), whereas in the younger group, it was near statistical significance (*p* = 0.05).

No statistically significant difference was observed for the serum concentrations of total protein, total cholesterol, thyroxin-binding prealbumin (TBPA), 25(OH)D, hemoglobin levels, or white blood cells between the FA group and the controls, either in the younger or in the older age group (*p* > 0.05).

A total of 21 children presented symptoms related to the gastrointestinal (GI) tract (diarrhea, constipation, abdominal pain), while 24 children were free from any GI symptoms, having the diagnosis of their allergy based on other symptoms only (such as atopic dermatitis or bronchial asthma). Short stature (hSDS < −2 SD) was found in 6 patients (28.5%) with GI symptoms and in 4 subjects with other symptoms (25%, *p* = 0.3). Underweight (BMI < −2SD) was also reported with a similar prevalence in children, either with or without gastrointestinal symptoms (20.8% vs. 23.8%, respectively, *p* = 0.8). No differences were found in the values of nutritional laboratory parameters in relation to the clinical symptoms (*p* > 0.05). The corresponding results are presented in Table 4.

## 5. Discussion

The results of our study show that food allergy, diagnosed in early childhood, is, in general, not associated either with heavily impaired physical development or with overt abnormalities in biochemical markers of the nutritional status at the time of diagnosis. Nevertheless, one of the more important observations in our study revealed that the patients under 30 months of age with newly diagnosed food allergies had been more susceptible to underweight. The observed difference did not apply to the older patients. That phenomenon possibly reflected the more severe impact of food allergy on child development in case of the early onset of allergy, which was also suggested in previous studies [9,10,11].

In the actual literature reports, body mass deficiency is frequently described in infants with cow’s milk allergy at diagnosis [12]. Among our patients from the younger age group, allergy to cow’s milk protein concerned approximately half of the group. The CAMEL study showed that the length and weight deficit affected infants with cow’s milk allergy and the potential catch-up for both body length/height and weight while following an elimination diet [9]. Additionally, Isolauri et al. [13] highlighted poor growth, in particular short stature, as a problem observed in children with IgE-mediated allergy to cow’s milk. In another study performed in children with atopic dermatitis, it was shown that more children in a food-restricted group presented with growth delay or underweight and also revealed lower head circumference [14]. No increased prevalence rate of growth deficiency was observed in our study of children with food allergies regardless of their age.

Dietary management plays a major role in the prevention and treatment of food allergy. The diagnosis of food allergy should be followed by the exclusion of allergenic foods from the diet. Cow’s milk allergy requires strict adherence to a prescribed elimination diet, often throughout the first year of life. This is a critical period for the development of food preferences and eating habits [15]. Children with milk allergies or multiple food allergies are at the greatest risk from avoidant/restrictive food intake disorder (ARFID), possibly contributing to certain nutrient deficiencies [16]. Flammarion et al. [6] found out in their study that children with IgE-mediated food allergies when eliminated three or more types of food, demonstrated significantly lower body weight- and height-for-age z-score. It seems that children at the time of diagnosis present nutritional deficiencies, resulting in weight (a predictor of short-term nutritional status) or, less often, height (a predictor of relatively long-term nutritional status) deficiencies. It is well documented that proper nutrition control in early life periods is of key importance for lifelong health status, particularly with regard to cardiovascular disease risk, bone health, and cognitive performance [10]. In addition to genetic predisposition, the development of allergies may also be influenced by the immaturity of the mucous membrane in the digestive system and by the underdeveloped GALT (gut-associated lymphoid tissue) [11].

According to the cited literature reports, it has been proven that children with food allergies are significantly more likely exposed to deficiencies of many nutrients than their healthy peers. They may develop a disease related to an exclusion diet and accompanied by an inflammatory process that may influence serum micronutrient concentrations in the body. Patients with either cow’s milk allergy or multiple food allergies are at high risk of ARFID, possibly contributing to certain nutrient deficiencies, which was confirmed using the results of numerous studies [13,17,18].

The currently known data used to assess the nutritional status of allergic patients at the time of diagnosis are rather limited. In our study, the laboratory parameters of nutritional statuses, such as the serum concentrations of total protein, total cholesterol, thyroxin-binding prealbumin (TBPA), vitamin D, hemoglobin level, and white blood cells, did not differ between the FA group and the controls. Similar observations were made by Low et al. [14], who showed that both hematological and biochemical profiles in children with atopic dermatitis were within their normal limits, regardless of whether food restrictions had been followed or not. At the same time, it was shown that although the albumin concentrations were normal, they were statistically significantly lower in patients on elimination diets.

The best markers of nutritional status include the proteins with a short half-life range as more sensitive indicators of protein synthesis and catabolism. Both thyroxin-binding-prealbumin (TBPA) and the retinol-binding protein (RBP) are early and sensitive indicators of malnutrition and can be used to monitor the efficacy of renutrition programs [18]. Among the older children in our study group, RBP was statistically significantly lower than in the controls, while that difference was borderline significant in the younger group. In a study by Canani et al. [19], normal RBP values were shown in patients with cow’s milk allergy; the same values remained within normal limits after the onset of an elimination diet but were not compared with the control group.

Numerous studies have thus far been published investigating the relationships between vitamin D and allergies. On the one hand, a link was postulated between vitamin D levels and the occurrence of allergic diseases, associating vitamin D deficiency with the risk of sensitization to food allergens [20]. Some available studies have confirmed the reduced supply of vitamin D and calcium in the diet and even reduced bone mineral density, especially in children with CMA [21,22]. However, that observation has not been confirmed in recent studies [16,23]. In our study, where the patients were evaluated at the time of allergy diagnosis, no differences were found in vitamin D concentrations between the study subjects and the controls.

To the best of our knowledge, our study was one of the few projects that assessed the nutritional status of pediatric patients with food allergies in relation to their clinical symptoms at the moment of diagnosis. Among the study group subjects, no differences were observed in the prevalence rates of height and weight deficiency, as well as in the laboratory indicators of the nutritional status in the patients, either with or without gastrointestinal involvement. Meyer et al. [24] also found that the allergic symptoms affecting the gastrointestinal tract, the respiratory tract, or the skin were not related to body growth parameters.

## 6. Limitations of the Study

A significant limitation of the study was the small size of the study group, especially of children under 30 months of age. In addition, no long-term observation was conducted on how the nutritional status and the examined biochemical parameters of the nutritional status were changing after the introduction of elimination diets. The study lacked some information on the diet, caloric content, eating habits, and the child’s diet (formula or breast milk). Additionally important was the lack of assessment of the nutritional status of the patients with non-IgE-mediated food allergies.

## 7. Conclusions

According to our findings, we may confirm that, regarding FA children under 30 months of age, one of the symptoms of food allergy may be body weight deficiency, while short stature is less common at the time of diagnosis. It seems, however, that pediatric patients with food allergy, when facing the onset of the elimination diet, do not present with laboratory parameters of malnutrition, except for lower RBP concentrations, observed especially in older children. In view of the cited literature, it is postulated to introduce an elimination diet program based on well-knit dietary advice and regular controls of nutritional markers, e.g., proteins with a short half-life such as RBP.

## Figures and Tables

**Table 1:** Results of specific IgE in the study group (*n* = 45).

Allergens	N (%)	Class of Allergy (n) Specific IgE Serum Concentration IU/mL			
		2 (0.7–3.49)	3 (3.5–17.4)	4 (17.5–49.9)	5 (50.0–99.9)
Egg white	36 (80%)	28	6	2	-
Egg yolk	15 (33.3%)	12	1	2	-
Cow’s milk	16 (35.5%)	8	4	3	1
Alpha -lactoglobulin	11 (24.4%)	8	1	2	-
Whey	8 (17.7%)	5	1	2	-
Casein	3 (6.6%)	2	-	1	-
Pork	4 (8.8%)	3	1	-	-
Wheat	3 (6.6%)	2	1	-	-
Rye	3 (6.6%)	2	1	-	-
Corn	1 (2.2%)	1	-	-	-
Rice	1 (2.2%)	1	-	-	-
Soybean	1 (2.2%)	-	-	1	-

**Table 2:** Comparison of anthropometric and laboratory parameters between the allergic patients (FA1) and the healthy controls (C1), aged ≤ 30 months of life.

Parameter	FA1 [*n* = 19]	C1 [*n* = 21]	*p* Value
Age (months)	16.2 ± 7.2	18.4 ± 8.2	0.3
hSDS	0.41 ± 2.2	−0.48 ± 1.4	0.1
BMI Z-score (kg/m^2^)	−0.75 ± 1.4	−0.4 ± 1.4	0.4
WBC (10^3^/µL)	9.38 ± 3.0	9.17 ± 2.0	0.7
Hgb (g/L)	11.77 ± 0.8	11.5 ± 1.0	0.5
TC (mg/dL)	143.9 ± 37.3	152.2 ± 27.1	0.5
Albumin (g/L)	43.83 ± 2.7	41.99 ± 2.8	0.9
Ferritin (µg/L)	35.0 ± 20.0	52.57 ± 39.5	0.1
25(OH)D (ng/mL)	35.52 ± 11.7	37.49 ± 17.3	0.7
RBP (mg/L)	18.59 ± 4.2	21.58 ± 5.1	**0.05**
TBPA (mg/L)	0.09 ± 0.01	0.1 ± 0.03	0.8

FA—food allergy patients, C—controls, TBPA—thyroxin-binding prealbumin, RBP—retinol-binding protein, WBC—white blood cells, TC—total cholesterol, Hgb—hemoglobin.

**Table 3:** Comparison of anthropometric and laboratory parameters between the allergic patients (FA2) and the healthy controls (C2), aged > 30 months of life.

Parameter	FA2 [*n* = 26]	C2 [*n* = 12]	*p* Value
Age (y)	50.0 ± 13.1	47.2 ± 10.4	0.5
hSDS	−0.47 ± 1.1	−0.15 ± 1.4	0.4
BMI Z-score (kg/m^2^)	−0.34 ± 0.9	0.02 ± 0.9	0.2
WBC (10^3^/µL)	9.34 ± 5.2	8.9 ± 2.5	0.7
Hgb (g/dL)	12.47 ± 0.9	12.38 ± 0.56	0.7
TC (mg/dL)	149.0 ± 36.4	172.3 ± 21.8	0.07
Albumin (g/L)	44.2 ± 5.1	44.16 ± 1.8	0.9
Ferritin (µg/L)	33.0 ± 19.8	41.4 ± 24.3	0.3
25(OH)D (ng/mL)	28.41 ± 8.8	27.47 ± 12.1	0.7
RBP (mg/L)	15.85 ± 3.1	18.52 ± 3.8	**0.02**
TBPA (mg/L)	0.09 ± 0.01	0.1 ± 0.02	0.2

FA—food allergy patients, C—controls, TBPA—thyroxin-binding prealbumin, RBP—retinol-binding protein, WBC—white blood cells, TC—total cholesterol, Hgb- hemoglobin.

**Table 4:** Nutritional laboratory parameters in the FA subjects, divided according to the presence of gastrointestinal symptoms.

Parameter	GI Involvement [*n* = 21]	Other Symptoms [*n* = 24]	*p* Value
WBC (10^3^/µL)	9.51 ± 4.41	9.18 ± 4.44	0.7
Hgb (g/dL)	12.26 ± 0.97	12.2 ± 0.97	0.5
TC (mg/dL)	146 ± 32.6	148.5 ± 42.1	0.8
Albumin (g/L)	43.6 ± 5.1	44.5 ± 2.9	0.5
25(OH)D (ng/mL)	29.6 ± 11.9	32.9 ± 8.2	0.3
RBP (mg/L)	16.2 ± 2.8	17.9 ± 4.6	0.13
TBPA (mg/L)	0.09 ± 0.01	0.1 ± 0.02	0.9

## Data Availability

The statistical analysis and database used to support the findings of this study may be released upon request by the Medical University of Silesia, Department of Pediatrics, via contact with the corresponding author.
